# Role of Melatonin in Angiotensin and Aging

**DOI:** 10.3390/molecules26154666

**Published:** 2021-07-31

**Authors:** Ahmet Ozer Sehirli, Serkan Sayıner, Ugochukwu Chukwunyere, Nedime Serakinci

**Affiliations:** 1 Department of Pharmacology, Faculty of Dentistry, Near East University, North Cyprus, Mersin 10, Nicosia 99138, Turkey; 2 Department of Biochemistry, Faculty of Veterinary Medicine, Near East University, North Cyprus, Mersin 10, Nicosia 99138, Turkey; serkan.sayiner@neu.edu.tr; 3 Department of Pharmacology, Faculty of Pharmacy, Near East University, North Cyprus, Mersin 10, Nicosia 99138, Turkey; ugochukwu.chukwunyere@neu.edu.tr; 4 Turkish Republic of Northern Cyprus Presidency, North Cyprus, Mersin 10, Nicosia 99138, Turkey; 5 Faculty of Medicine, Cyprus International University, Haspolat, North Cyprus, Mersin 10, Nicosia 99138, Turkey

**Keywords:** aging, angiotensin, inflammation, melatonin, oxidative stress

## Abstract

The cellular utilization of oxygen leads to the generation of free radicals in organisms. The accumulation of these free radicals contributes significantly to aging and several age-related diseases. Angiotensin II can contribute to DNA damage through oxidative stress by activating the NAD(P)H oxidase pathway, which in turn results in the production of reactive oxygen species. This radical oxygen-containing molecule has been linked to aging and several age-related disorders, including renal damage. Considering the role of angiotensin in aging, melatonin might relieve angiotensin-II-induced stress by enhancing the mitochondrial calcium uptake 1 pathway, which is crucial in preventing the mitochondrial calcium overload that may trigger increased production of reactive oxygen species and oxidative stress. This review highlights the role and importance of melatonin together with angiotensin in aging and age-related diseases.

## 1. Introduction

Aging is a gradual process in an organism characterized by a progressive decline of physiological functions, including homeostatic mechanisms, at all levels [1]. The aging process may lead to an increased risk of fatigue, disease, and death [2]. In recent years, researchers have become increasingly interested in finding ways of slowing the aging process [3,4,5]. However, it is important to note that aging is not a disease. Angiotensin has numerous functions including blood pressure control and body fluid and electrolyte balance, and can contribute to aging by stimulating the formation of free radicals that provoke mitochondrial disruption and cellular damage [6,7,8]. The functional integrity of the cell structure can be compromised by the presence of these free radicals in the cell membranes. Melatonin, a hormone with a strong antioxidant effect, helps preserve the cell membrane by neutralizing the highly reactive free radicals that damage the cells [9]. Therefore, elucidating the factors and mechanisms of aging may help ease age-related complications. Herein, we review available studies on the current role and importance of melatonin and angiotensin in aging and age-related diseases.

## 2. Mechanism of Aging

Aging is a universal phenomenon that begins in early adulthood and continues throughout life [10,11]. The cellular and molecular hallmarks of aging have been described, and the interconnection between these aging hallmarks helps to reduce damage in the organism [10]. Tissue aging is associated with enhanced senescent cells at the cellular level while early aging is associated with more senescent cells, trans-differentiated cells, and inflammatory cells [12,13]. Genomic instability, however, contributes significantly to the advancement of cell aging, and age-related cancer, as well as neurodegenerative and vascular diseases [14,15]. Some molecules dubbed as ‘aging genes’ and ‘longevity genes’ may be implicated in the process of aging. However, the roles played by these molecules in physiological and pathological aging are yet to be fully elucidated [16,17]. Furthermore, impairment of the defense and repair genes observed at the molecular level may be involved in the entire process of aging. 

There is significant evidence which suggests that telomeres are the most common targets for cell senescence and chronic DNA damage response in aging, while failure to maintain a minimum telomere length triggers cell replicative senescence [18,19]. Differences in telomere length have been linked to different diseases such as dyskeratosis congenita and liver cirrhosis. These diseases have been suggested as being associated with telomerase gene mutations that replace lost telomeres [10]. Investigations aimed at identifying why the shortest telomeres are chosen for repair have revealed that accumulated telomeric repeat-containing RNA (TERRA) forms RNA–DNA hybrids (R-loops) at short telomeres, and that high TERRA levels and R-loops at short telomeres result from the failure of RNA degradation at specific sites. These findings suggest that the rate of replicative senescence is determined by the telomere length-dependent control of TERRA R-loops [20,21,22].

Aging can also be triggered by loss of lysosomal dysfunction, which is closely linked with the process of autophagy, an intracellular self-cleansing mechanism that degrades unwanted or damaged components that accumulate within the cell. Accumulation of potentially toxic molecules such as denatured RNA may lead to permanent loss of distinct cell functions, which is indicative of cellular degeneration [23,24,25,26,27]. Emerging evidence has suggested that defective stem cell autophagy contributes to aging and degenerative diseases characterized by a decline in stem cell regenerative ability [28]. Dysfunctional stem cells in aged mice maintained a low metabolic state and high autophagy levels with long-lasting robust regenerative ability, suggesting that autophagy may slow cellular metabolism to sustain stem cell viability and regenerative ability in aging [29].

## 3. Free Radicals in Aging Process

Free radicals are highly reactive atoms or molecules with one or more unpaired electrons in their outer shell [30]. According to the ‘free radical theory of aging’, the deleterious effects of free radicals on cell components and connective tissues can lead to aging and age-related degenerative diseases [31,32]. When oxygen molecules split into single atoms with unpaired electrons, they form unstable free radicals that seek to bind with other atoms or molecules. Superoxide, alkoxyl radicals, hydroxyl radicals, as well as hydrogen peroxide are reactive radical and non-radical oxygen derivatives described as reactive oxygen species (ROS). Reactive nitrogen species (RNS) are a group of molecules derived from nitric oxide and superoxide through the enzymatic activity of inducible nitric oxide synthase and NADPH oxidase, respectively [33,34,35]. These radicals are generated in cells by gaining or losing a single electron, thus acting as oxidants or reductants [36]. Oxidants are produced as a result of regular cellular utilization of oxygen and several cytosolic enzyme activities in the mitochondria.

Superoxide anions generated during oxidative phosphorylation in the mitochondria are rapidly converted to hydrogen peroxide by superoxide dismutase (SOD). In specific circumstances marked by the presence of transition metals, highly reactive hydroxyl ion can be formed through the Haber–Weiss or Fenton-type reaction [30]. These hydroxyl radicals have high reactivity (especially with phospholipids, a main component of the cell membrane) [37,38,39]. Alkoxyl radicals generated from the reduction of peroxides are less reactive than hydroxyl radicals and are substantially more reactive than peroxyl radicals. As a result, they are particularly suitable for assessing the efficiency of antioxidants as well as the reactivity of ROS with any radical species [40]. However, the reaction between nitric oxide radicals and superoxide produces peroxynitrite, a known cytotoxic compound and biomarker of nitro-oxidative stress [41].

An imbalance characterized by the enhanced generation of ROS/RNS and diminished antioxidant defenses triggers oxidative stress (OS), cell damage, and ultimately aging [42]. In addition, ROS/RNS are involved in cell signaling, the extraction of energy from organic compounds, and immune defense [43]. Regardless of their source, ROS/RNS can cause oxidative changes by reacting with several biologically active molecules including DNA, proteins, carbohydrates, and lipids. Hence, ROS/RNS can serve as potent biomarker of OS [44,45,46].

### 3.1. Advanced Glycation End Products

Several oxidative modifications in lipids and nucleic acids have been described, including the oxidative breakdown of free amino groups of proteins to produce potentially toxic substances that have been linked to aging [47]. The reactive carbonyl groups of a reducing sugar react with nucleic acids, lipids, and free amino groups (especially of arginine and lysine residues) to form advanced glycation end products (AGEs) such as glucosepane, carboxyethyl-lysine, and hydroimidazolone [48,49]. The primary source of AGEs in humans (including endogenous AGEs) are continuously generated in the body, in particular in diabetics with impaired glucose metabolism and exogenous AGEs derived from smoking and dietary foods processed at high temperature [41]. Poly-unsaturated fatty acids (PUFAs), such as arachidonic and linoleic acids, are important targets for lipid peroxidation induced by hydroxyl and peroxyl radicals. Several reactive aldehydes such as malondialdehyde (MDA) and trans-4-hydroxy-2-nonenal (4-HNE) are produced during the lipid oxidation process [49]. It has been reported that the increased consumption of dietary AGEs can cause the buildup of AGEs which may have a negative impact on the body due to their ability to enhance OS and inflammation [48]. AGEs increase inflammation and OS by binding to the receptor for advanced glycation end products (RAGE), which are expressed in the heart, skeletal muscle, skin, and lungs. The binding of AGEs to these receptors promotes the activation of the nicotinamide adenine dinucleotide phosphate (NADPH) oxidase and nuclear factor-kappa B (NF-κB) pathway, increases the production of ROS, and also prolongs cellular dysfunction and tissue damage [50].

### 3.2. Antioxidants

The antioxidant defenses neutralize the deleterious effects of ROS and RNS formed by a variety of exogenous and endogenous agents including NADPH oxidase, a known source of radical superoxide anion [42]. A widely proposed approach for lowering ROS involves scavenging free radicals and boosting antioxidant defenses. Antioxidants serve as free radical scavengers, preventing oxidative changes that can cause a variety of diseases [31]. The antioxidant defense system consists of exogenous antioxidants derived from diets and endogenous antioxidants which may be enzymatic or nonenzymatic compounds located in the cytoplasm [32]. Several antioxidant enzymes including catalase, SOD, and certain peroxidases convert ROS into more stable molecules in eukaryotic cells through a complex chain reaction [51]. SOD is believed to be one of the most potent intracellular enzymatic antioxidants that catalyze the dismutation of superoxide radicals. The three forms of SOD isoenzymes found in humans include cytosolic copper-zinc-dependent SOD (CuZn-SOD), extracellular SOD (EC-SOD), and mitochondrial manganese-dependent SOD (Mn-SOD), which plays an important role in detoxifying the free radical superoxide formed during mitochondrial respiration [52]. Other biologically active antioxidant enzymes such as glutathione peroxidase, catalase, and glutathione reductase spontaneously convert hydrogen peroxide into water and oxygen [51].

Nonenzymatic antioxidants such as thioredoxin, tocopherol (vitamin E), and ascorbic acid (vitamin C), including some essential components of the antioxidant defense systems such as selenium and NADPH, also act as scavengers of ROS. The most frequently used antioxidant vitamins, tocopherol and ascorbic acid, are believed to reduce or preventive oxidative damage caused by ROS [53]. Melatonin acts as a direct scavenger to detoxify ROS/RNS, thereby protecting different biomolecules from free radical-induced oxidative and nitrosative damage [54]. 

A progressive decline in ROS scavengers shifts aged cells towards a pro-oxidant profile which may result in the inability to buffer ROS formed in both normal and pathologic conditions [55]. Studies have shown that increased mitochondrial ROS levels play a mechanistic role in the aging of genetically altered animals [56,57] and that animals deficient in SOD show mitochondrial dysfunctions which trigger oxidative damage and other traits akin to early aging [58,59]. Mice deficient in cytoplasmic copper/zinc SOD are unable to detoxify ROS, and so they have high ROS levels but show a normal lifespan [60]. Mice that overexpress mitochondrial catalase, on the other hand, are less susceptible to OS and live longer [61]. In worms, high cytoplasmic or mitochondrial ROS levels are linked to shorter and longer lifespans, respectively [62,63]. When an antioxidant reacts with a free radical, an oxidized form of the antioxidant is formed, which may also enhance the activity of the endogenous defense systems [64]. Therefore, several antioxidants have physiologically lowering mechanisms and, in some instances, their oxidized forms may induce the effect of hormesis as an adaptive response to increase cellular defense by activating the endogenous antioxidant defense systems [65]. However, there are contradictory reports on the association between supplementation with natural or synthetic antioxidants and desired health benefits [66,67,68].

## 4. Angiotensin and the Aging Process

**Figure 1:**
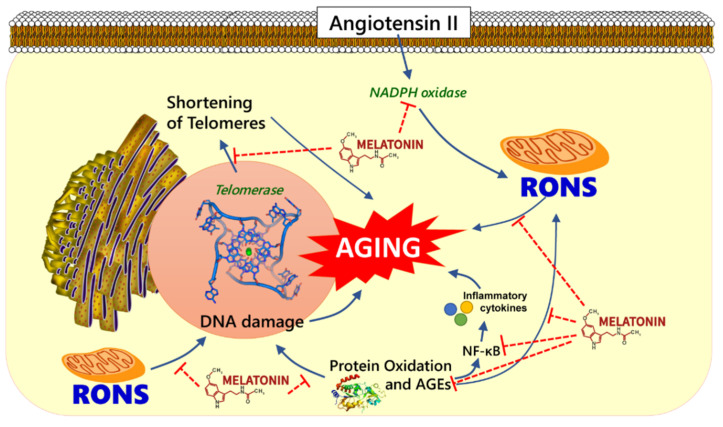
Schematic demonstration of potential antiaging mechanism of melatonin. Melatonin could play a role on aging hallmarks e.g., melatonin may attenuate angiotensin-induced oxidative damage by suppressing the production of reactive oxygen and nitrogen species (RONS), inflammatory cytokines, advanced glycation end products (AGEs) and preventing telomere shortening.

The renin-angiotensin-aldosterone system (RAAS) has been shown to influence longevity in different animal species including rodents and nematodes [69]. Studies have shown that an increased activation of intrarenal RAAS triggers the enhanced production of ROS, glomerular sclerosis, tubulointerstitial fibrosis, and altered sodium levels [70,71]. Angiotensin II (Ang II), an active component of RAAS, acts through the angiotensin II type 1 (AT1) receptor to activate NADPH oxidase which triggers the production of ROS and OS damage [72] (Figure 1). Because of its pro-oxidant properties, Ang II may play a role in organ aging since Ang II-induced ROS promotes early vascular aging associated with structural and functional alterations in blood vessels that contribute to age-related vascular disorders [73]. 

Benigni et al. (2009) studied the effects of disrupted *Agtr1a* gene encoding AT1a, an AT1 receptor isoform in mouse and reported that AT1a knockout mice had fewer vascular disorders and outlived their genetically matched wild-type counterparts [74]. RAAS inhibition has been shown to be crucial in preventing cardiac remodeling and heart failure. Inhibition of AT1 receptors and RAAS reverses age-related fibrosis, severe myocardial hypertrophy, and cerebrovascular damage caused by the buildup of oxidative substances in the blood vessels of animals [75,76,77,78]. The serum antibodies that target the AT1 receptor have been shown to contribute to vascular allograft rejection in kidney transplant patients; however, treatment with losartan, an AT1 receptor antagonist, blocked the agonistic AT1 receptor response to the antibodies [79].

RAAS also contributes to the pathophysiology of other age-related diseases such as dementia, cancer, osteoporosis, and diabetes [80]. RAAS plays a role in the development of insulin resistance, which is a common feature of type 2 diabetes [81]. Results from different randomized clinical trials showed that inhibition of RAAS by angiotensin converting enzyme inhibitors and AT1 receptor blockers improves insulin sensitivity and lowers the incidence of type 2 diabetes [82]. Insulin signaling is regulated by Ang II-induced AT1 receptor activation. Activation of AT1 receptor increases the growth and proliferative actions of insulin while inhibiting its metabolic actions [81,83].

AT1 receptors are present in various human cancer cell lines, including prostate and pancreatic cell lines [84,85]. Previously, it has been reported that the proliferation of tumor cells embedded in AT1a receptor-deficient animals decreased, as well as tumor-related angiogenesis [86]. In a mouse model of lung adenocarcinoma, Ang II increased the proliferation of myeloid progenitor cells in the spleen, thus causing tumor-associated macrophages to enhance tumor growth [87]. Therefore, RAAS signaling contributes to tumor growth by promoting tumor cell proliferation, tumor-linked macrophage expansion, and angiogenesis.

The genetic alteration of the AT1a receptor was found to increase the expression of nicotinamide phosphoribosyl transferase (NAMPT) and Sirtuin-3 (SIRT3) in the kidneys of aged mice [74]. Increased NAMPT expression in a nutrient-deprived medium causes mitochondrial accumulation of nicotinamide adenine dinucleotide that activates SIRT3 in the mitochondria. Therefore, RAAS inhibition prolongs lifespan by reducing OS and increasing the expression of anti-apoptotic genes [88].

## 5. Melatonin in Aging 

Melatonin is a hormone with a strong antioxidant effect and is found naturally in several living organisms including human beings [89,90]. Melatonin helps in the preservation of cell membrane by neutralizing highly toxic hydroxyl radicals which induces lipid peroxidation. In addition to neutralizing the radical superoxide anion, peroxynitrite anion, and hydrogen peroxide, melatonin enhances gene expression for glutathione peroxidase and other antioxidant enzymes [9,91,92]. Melatonin’s metabolites, such as N^1^-acetyl-N^2^-formyl-5-methoxykynuramine and N^1^-acetyl-5-methoxykynuramine, have potent antioxidant and anti-inflammatory effects [93,94,95]. Studies have shown that melatonin stimulates antibody production and modulate immune functions including anti-tumorigenic defense. Therefore, melatonin acts as an immunomodulator in both physiological and pathological conditions [95,96,97].

Disruption of the rhythmic release of melatonin has been previously linked to the severity of chronic kidney disease (CKD) [98]. Early-stage CKD patients have been reported to have increased oxidation of DNA, proteins, and lipids, which may contribute to organ damage [99]. The disease progress of CKD is associated with increased OS and inflammation, which promotes the buildup of uremic toxins that exacerbates renal failure [100,101]. CKD patients undergoing hemodialysis are also at a greater risk of inflammation and mortality [102]. Furthermore, peritoneum fibrosis is caused by OS and inflammatory responses during peritoneal dialysis [103]. Melatonin improves CKD outcomes by inhibiting RAAS [104], and long-term exogenous melatonin treatment enhances the expression of vasoprotective biomarkers while decreasing inflammation and OS [105]. Melatonin and vitamin D interact through different pathways to support and maintain functional mitochondrial integrity. Transcription factors such as runt-related transcription factor 2 (Runx2) and vitamin D receptor (VDR) regulate the expression of target genes involved in osteoblast differentiation and bone formation [106,107]. According to Fang et al. (2020) the indirect regulation of Runx2 by melatonin can be attributed to its ability to bind directly to the VDR [106]. Prado et al. (2018) reported that the positive impact of melatonin on the regulation of the VDR is mostly due to its ability to bind 1,25-dihydroxyvitamin D3 (VD3) to regulate calcium mobilization, which indirectly affects osteoblast differentiation and bone formation [108]. Moreover, VD3 plays an antiaging role by activating the nuclear factor-erythroid factor 2-related factor 2 (Nrf2) that regulates the expression of antioxidant defense genes [109]. The immunomodulatory and anti-proliferative effects of vitamin D have been demonstrated in both in vivo and in vitro studies. Activation of the VDR stimulates the expression of DNA damaged-induced transcription 4, which promotes abnormal cell differentiation by inhibiting the activation of mammalian target of rapamycin (mTOR) via the tuberous sclerosis complex (TSC) signaling pathway [110,111,112]. mTOR is known to regulate proliferation, protein synthesis, growth, and survival [113]. However, studies have shown that by blocking mTOR signaling, rapamycin promotes mitophagy and attenuates apoptosis and generation of ROS in the cells [114,115].

Melatonin exerts indirect antiarrhythmic effects through its cardiorenal protective actions. This was illustrated using rat model of unilateral ureteral-obstruction (UUO). Results from the study showed that melatonin’s protective effect against myocardial remodeling contributed to its action against ventricular fibrillation induced by UUO. By increasing the heat shock protein 70 (Hsp70) and VDR and decreasing AT1, melatonin reduced fibrosis, oxidative stress, and myocardial cell death [108]. In addition, Gubin et al. (2016) studied the effects of melatonin in age-dependent alterations in heart rhythms and reported that night-time administration of melatonin attenuated morning rise in heart rate in both normotensive and hypertensive subjects [116].

In most vertebrates, melatonin synthesis declines with age. This decline could be attributed to the decrease in the number of β-adrenergic receptors in the pineal gland as well as a decrease in the expression of AANAT/SNAT genes [117,118]. Old cells generate more RNS/ROS, but the endogenous antioxidative effect of melatonin counteracts RNS/ROS production in aging cells [119]. Pinealectomy-induced suppression of melatonin enhanced aging due to the buildup of reactive molecules [120]. In contrast, transplanting young pineal glands into aged animals and exogenous melatonin supplement significantly increased the lifespan of animal models [121]. Melatonin has been shown to reduce telomerase activity in cultured cancer cells and prevent metastases in an athymic animal model [122]. These findings support the view that melatonin inhibits thioretinaco ozonide loss from mitochondria during carcinogenesis by preventing mPTP pore opening [123]. Akbulut et al. (2009) studied the effects of melatonin on age-related variations in telomerase activity and the rate of cellular proliferation in gastric mucosa. They concluded that melatonin slows gastric mucosal aging by stimulating telomerase activity and inhibiting lipid peroxidation and cellular proliferation [124].

Melatonin and angiotensin interact at different levels from the site of synthesis to the sites of action. The pineal and pituitary glands have been shown to have high Ang II-forming activities due to the presence of human chymase in the brain, thus suggesting the presence of a local RAAS in the pineal gland [125]. A precursor of RAAS, angiotensinogen is present at a very low level in the pineal glial cells, whereas AT1b receptors are expressed in pinealocytes [126]. The synthesis of melatonin from tryptophan is a four-step process that involves a number of enzymes including tryptophan hydroxylase (TPH), the rate-limiting enzyme in serotonin biosynthesis. Ang II modulates the synthesis and activities of TPH, by acting on AT1 receptors in pinealocytes [127]. Ji et al. (2016) studied the protective effects of melatonin on podocytes of diabetic nephropathy model animals. Results from that study showed that treatment with melatonin suppressed angiotensin II-induced podocyte damage by improving mitochondrial activity [128].

Melatonin acts through the MT1 and MT2 receptors to induce vasoconstriction and vasodilation, respectively [129]. However, a significant decrease in melatonin levels has been observed in cardiovascular diseases. These findings suggest that angiotensin and melatonin have counteractive actions in the cardiovascular system, possibly due to the direct free radical scavenging, antioxidant activity, and sympatholytic effect of melatonin. Yang et al. (2021) reported that melatonin may protect against cardiac hypertrophy by activating the mitochondrial calcium uptake 1 (MICU1) pathway to produce more MICU1, an important molecule that maintains homeostasis during Ang-II-induced stress [130]. Considering the role of angiotensin in aging, melatonin modulates both MICU1 and angiotensin levels, and this may contribute to its protective effect [1,130,131]. 

## 6. Conclusions

Free radicals derived from either endogenous or exogenous sources can cause oxidative modifications of the major biological macromolecules, leading to increased oxidative stress. Angiotensin triggers the production of free radicals by activating the angiotensin II type 1 receptors. Free radical-induced oxidative stress is presumed to contribute to aging and several diseases associated with aging. Melatonin can enhance the expression and activities of some antioxidative enzymes, block a possible pro-oxidative enzyme pathway, bind metals that release free radicals, stimulate the immune system, and ultimately prolong longevity (Figure 1).

## Data Availability

Not applicable.
